# SARS-CoV-2 induces Alzheimer’s disease–related amyloid-β pathology in ex vivo human retinal explants and retinal organoids

**DOI:** 10.1126/sciadv.ads5006

**Published:** 2025-07-04

**Authors:** Sean J. Miller, Rahul M. Dhodapkar, Hande Eda Sutova, Yao Xue, Seunghoon Lee, Robert Logan, Chongzhao Ran, Sagar Bhatta, Ashley Gomm, In Gyoung Ju, Michael Heyang, Rayyan Y. Darji, Marcello DiStasio, Rudolph E. Tanzi, Can Zhang, Z. Jimmy Zhou, Brian P. Hafler

**Affiliations:** ^1^ Department of Ophthalmology and Visual Science, Yale University School of Medicine, New Haven, CT, USA.; ^2^ Department of Biology and Biotechnology, School of Science and Technology, Endicott College, Beverly, MA, USA.; ^3^ Martinos Center for Biomedical Imaging, Massachusetts General Hospital and Harvard Medical School, Boston, MA, USA.; ^4^ Genetics and Aging Research Unit, McCance Center for Brain Health, Massachusetts General Institute for Neurodegenerative Disease, Department of Neurology, Massachusetts General Hospital and Harvard Medical School, Charlestown, MA, USA.; ^5^ Department of Pathology, Yale University School of Medicine, New Haven, CT, USA.; ^6^ Broad Institute of MIT and Harvard, Cambridge, MA, USA.

## Abstract

While the etiology of Alzheimer’s disease remains unknown, there is growing support for the amyloid-β antimicrobial hypothesis. Amyloid-β, the main component of amyloid plaques in Alzheimer’s disease, has been shown to be generated in the presence of microbes. Entrapment of microbes by aggregated amyloid-β may serve as an innate immune response to pathogenic infections. To understand the association of amyloid-β plaques and pathogenic infections in the central nervous system, we obtained viable short-interval postmortem human retinal tissue and generated human retinal organoids that contain electrophysiologically active neurons. Here, we demonstrate that severe acute respiratory syndrome coronavirus 2 (SARS-CoV-2) induces amyloid-β extracellular protein aggregates in human retinal explants and retinal organoids. Last, pharmacological inhibition of neuropilin-1 resulted in reduced amyloid-β deposition in human retinal explants treated with SARS-CoV-2 Spike 1 protein. These results suggest that Spike 1 protein, during infection with SARS-CoV-2, can induce amyloid-β aggregation, which may be associated with the neurological symptoms experienced in COVID-19.

## Teaser

SARS-CoV-2 induces amyloid-β pathology in the central nervous system.

## INTRODUCTION

Alzheimer’s disease is a leading cause of dementia, characterized by amyloid-β plaques and the entanglement of tau (*1*, *2*). Since Alois Alzheimer first described Alzheimer’s disease in 1906, the scientific community has sought therapeutic interventions and elucidation of the disease pathophysiology (*3*). Alzheimer’s disease is mainly sporadic, with less than 5% of all cases being represented by familial mutations in the amyloidogenic pathways: amyloid-β precursor protein (APP), presenilin-1 (PSEN1), and PSEN2 (*4*). One hypothesis associated with the etiology of Alzheimer’s disease is that amyloid-β aggregation serves as an innate immune protective mechanism against microbial infection (*5*–*7*). The antimicrobial hypothesis of amyloid-β has been supported by the production of amyloid-β in response to bacteria, viruses, parasites, and fungal infections (*7*). Similar to Alzheimer’s disease, aging correlates with a loss in the integrity of the blood-brain barrier, a multicomponent tissue interface system that is typically protective but can allow pathogens to infiltrate the brain with age (*8*–*10*). With the COVID-19 pandemic, caused by the severe acute respiratory syndrome coronavirus 2 (SARS-CoV-2) virus, there is a need to understand the aging central nervous system’s (CNS) response to viral infection (*11*).

SARS-CoV-2 has infected more than 750 million people, leading to COVID-19 and more than 7 million fatalities (*12*). The short-term effects of infection include flu-like symptoms accompanied by a biological cytokine storm that ranges in severity and anatomical location (*13*, *14*). SARS-CoV-2 can bind to neurons via neuropilin-1 (NRP1), angiotensin-converting enzyme 2 (ACE2), and several other receptors (*15*–*19*). The binding of SARS-CoV-2 Spike 1 protein can induce an innate immune response that results in the production and release of antimicrobial peptides (*20*, *21*). SARS-CoV-2 has been shown to infect neurons and alter amyloidogenic enzymatic processing (*18*, *22*–*24*). SARS-CoV-2 Spike protein has been reported to up-regulate amyloid-β through modulating γ-secretase (*22*). One study demonstrated the affinity of amyloid-β to the Omicron and Alpha variants of the SARS-CoV-2 Spike 1 protein (*25*). Two recent studies detected SARS-CoV-2 in the postmortem human retina from patients with COVID-19, further illustrating SARS-CoV-2 infection in the CNS (*26*, *27*). While many of the features of the short-term illness have been elucidated, the long-term consequences of SARS-CoV-2 infection such as potential exacerbation of dementia and memory loss have not been well defined (*10*, *21*, *28*–*30*).

The amyloid-β plaque burden in the brain of patients with Alzheimer’s disease is observed in the human retina, which is part of the CNS (*31*–*33*). The human retina is a clinically and visually accessible area of the CNS where one can evaluate Alzheimer’s disease pathophysiology (*33*). In this study, we detected elevated amyloid-β in postmortem human retinal tissue from Alzheimer’s disease and COVID-19 donors. We validated this mechanism using ex vivo and in vitro assays with human postmortem retinas from short-interval autopsies and human retinal organoids. Blocking of NRP1 dampened amyloid-β accumulation induced by Spike 1 protein, identifying a possible new therapeutic target for SARS-CoV-2 in the CNS. Thus, increased amyloid-β in patients with COVID-19 may be associated with compromised memory and learning processes that can result in neurological impairment.

## RESULTS

### Short-interval human retinas display neural activity under patch-clamp recording

**Fig. 1.**
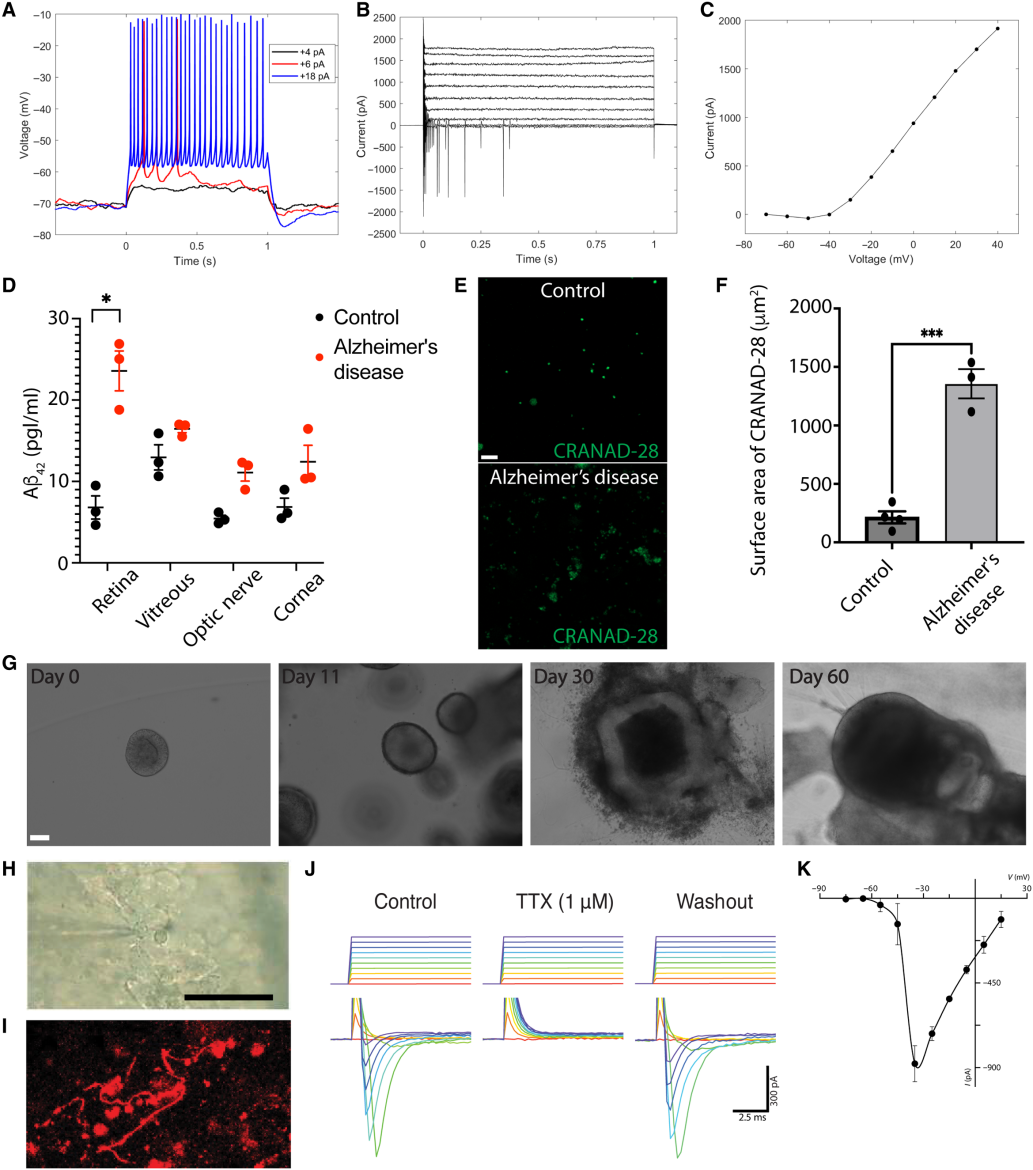
Retinas and retinal organoids contain active neurons with amyloid-β pathology. (**A**) Spikes evoked in a midget ganglion cell under patch clamp (current clamp) in whole-mount postmortem human retina, showing subthreshold (black trace), low suprathreshold (red), and large suprathreshold voltage responses (blue) to current injections of three different amplitudes (4, 8, and 18 pA). (**B**) Voltage-activated currents under voltage clamp from a holding potential of −80 mV to various depolarizing voltage steps (in 10-mV increments), showing leak-subtracted fast inward Na^+^ currents and sustained outward K currents. (**C**) *I*-*V* curve of K currents measured at the end of the pulses’ voltage clamp. (**D**) amyloid-β42 ELISA (Abcam) of regions of the human Alzheimer’s disease (*n* = 3) and control globes (*n* = 3; error bars denote SEM). (**E**) CRANAD-28 (green) labels amyloid-β in Alzheimer’s disease (*n* = 3) postmortem retinas as compared to age-matched controls (*n* = 4; scale bar, 50 μm). (**F**) CRANAD-28 surface area is significantly increased in the Alzheimer’s disease retina compared to controls (*P* < 0.0002; *n* = 6; error bars denote SEM). (**G**) Bright-field microscopy reveals the development of retinal organoids on days 0, 11, 30, and 60; scale bar, 50 μm. (**H**) Differential interference contrast micrograph of a surface-located cell recorded with Alexa Fluor 594 (red)–filled patch pipette (scale bar, 50 μm). (**I**) Two-photon image (maximum *z* projection) of the same retinal neuron in (H), showing developing neurites with Alexa Fluor 594. (**J**) Voltage-gated inward currents of human retinal organoids evoked by a series of voltage steps from −75 mV (200 ms long, 10 mV increment) were completely blocked by TTX (1 μM) and fully recovered after a 25-min washout. (**K**) Relationship between the peak Na current amplitude and the level of membrane potential in control solution (control; *n* = 2; error bars denote SDs).

To determine the viability of postmortem human retinas from rapid autopsies (<4 hours), we recorded from retinal ganglion cells in a flat-mount preparation. The human retinal cells generated robust spikes when stimulated with small depolarizing current pulses under current clamp (Fig. 1A). Figure 1A shows an example of voltage responses from an ON-midget ganglion cell to subthreshold and suprathreshold current pulses, displaying a spike rate that was proportional to the stimulus amplitude and reached a maximum value of 37 spikes/s (mean ± SD, 31 ± 5; *n* = 3) for an 18-pA current pulse. Under voltage clamp, the retinal cell exhibited fast inward Na^+^ currents and sustained outward K^+^ currents upon activation by voltage steps of incremental amplitudes (Fig. 1B). The repetitive fast inward currents, a sign of escaping from voltage clamp and space clamp, were consistent with the observation that the cell maintained intact dendrites and axon in the postmortem human retinal explant. Figure 1C shows the current-voltage relation (*I*-*V*) of the sustained outward K^+^ currents measured at the end of the pulses. Overall, the spiking behavior and the intrinsic currents of the cells suggested a neuronally active state of our postmortem human retinal explant preparations.

### Human Alzheimer’s disease retinas exhibit widespread amyloid-β deposits

To analyze for regional specificity of amyloid-β42 in human globes with Alzheimer’s disease, we performed enzyme-linked immunosorbent assay (ELISA) on the following anatomical regions: retina, vitreous, optic nerve, and cornea from human Alzheimer’s disease (*n* = 3) and age-matched controls (*n* = 3). We noted a statistically significant increase in the concentration of amyloid-β42 in Alzheimer’s disease human retinas when compared to the controls (*P* < 0.05; Fig. 1D). To show that the human retinas from postmortem donors with Alzheimer’s disease contained amyloid-β, we assessed rapid autopsy retinas from controls (*n* = 4) and Alzheimer’s disease (*n* = 3) for the presence of extracellular plaque formation using the small-molecule curcumin analog CRANAD-28 (*34*–*37*). CRANAD-28 does not require fixation, allowing for the detection, visualization, and quantification of amyloid-β plaques in human retinas. Treatment with CRANAD-28 revealed widespread amyloid-β plaques in Alzheimer’s disease retinas (*n* = 3) as compared to the age-matched controls (*n* = 4; Fig. 1, E and F). We confirmed these findings with an antibody for amyloid-β, 6E10, in postmortem human retinas with Alzheimer’s disease (fig. S1, A and B) (*38*). In addition, we observed astrogliosis of the nerve fiber layer of retinas derived from Alzheimer’s disease donors, in corroboration with prior studies (fig. S1C) (*39*). These data indicate the presence of amyloid-β deposits and neuropathology in postmortem human retinas from individuals with Alzheimer’s disease.

### Human retinal organoids contain electrophysiologically active neurons

Next, we generated human retinal organoids to study the effects of SARS-CoV-2 infection on the CNS. We differentiated and matured human-induced pluripotent stem cells into complex multicellular retinal organoids using an accelerated aging protocol that promotes the development of the retinal pigment epithelium, retinal ganglion cells, neuronal subtypes, and macroglia in a laminar architecture (*40*). At each developmental time point (days 0, 11, 30, and 60), we confirmed that the human retinal organoids maintained appropriate cytomorphology and architecture with bright-field microscopy (Fig. 1G). Throughout the timeline, key developmental milestones were achieved, such as embryoid bodies (day 0), neural spheres (day 11), retinal ganglion cells (day 30), and complex human retinal organoids (day 60; Fig. 1G). On day 60, we confirmed the presence of neurons in the human retinal organoids with the neuronal marker microtubule-associated protein 2 (MAP2; fig. S2). To confirm that day 60 human retinal organoids derived from Alzheimer’s disease human induced pluripotent stem cells (iPSCs), harboring familial genetic mutations in APP (Swedish) and PSEN1 (M146V), are capable of recapitulating disease features such as the production of amyloid-β species, we performed immunofluorescence using an antibody specific for amyloid-β and ELISA for amyloid-β (*41*, *42*) (fig. S3, A to C). We detected elevated levels of amyloid-β in the Alzheimer’s disease retinal organoids as compared to the controls using both immunofluorescence and ELISA (fig. S3, A to C).

We assessed whether the human retinal organoids were functional by examining membrane excitability using whole-cell voltage-clamp recordings from individual putative neurons (Fig. 1, H and I). Upon applying a series of depolarizing voltage steps (from −75 to 15 mV, in 10-mV increments), the recorded cells displayed large, transient inward currents (~1 nA at −35 mV), resembling neuronal sodium current (Fig. 1J). Complete blockade of the currents by tetrodotoxin (TTX; 1 μM), followed by washout and recovery, further confirmed that these currents were sodium currents, which are typically found in ganglion cells of normal retina (Fig. 1, J and K). These results show the presence of excitable retinal ganglion cells in the retinal organoids and functional human neurons.

### Single-cell RNA sequencing and immunofluorescence label neurons with SARS-CoV-2

**Fig. 2.**
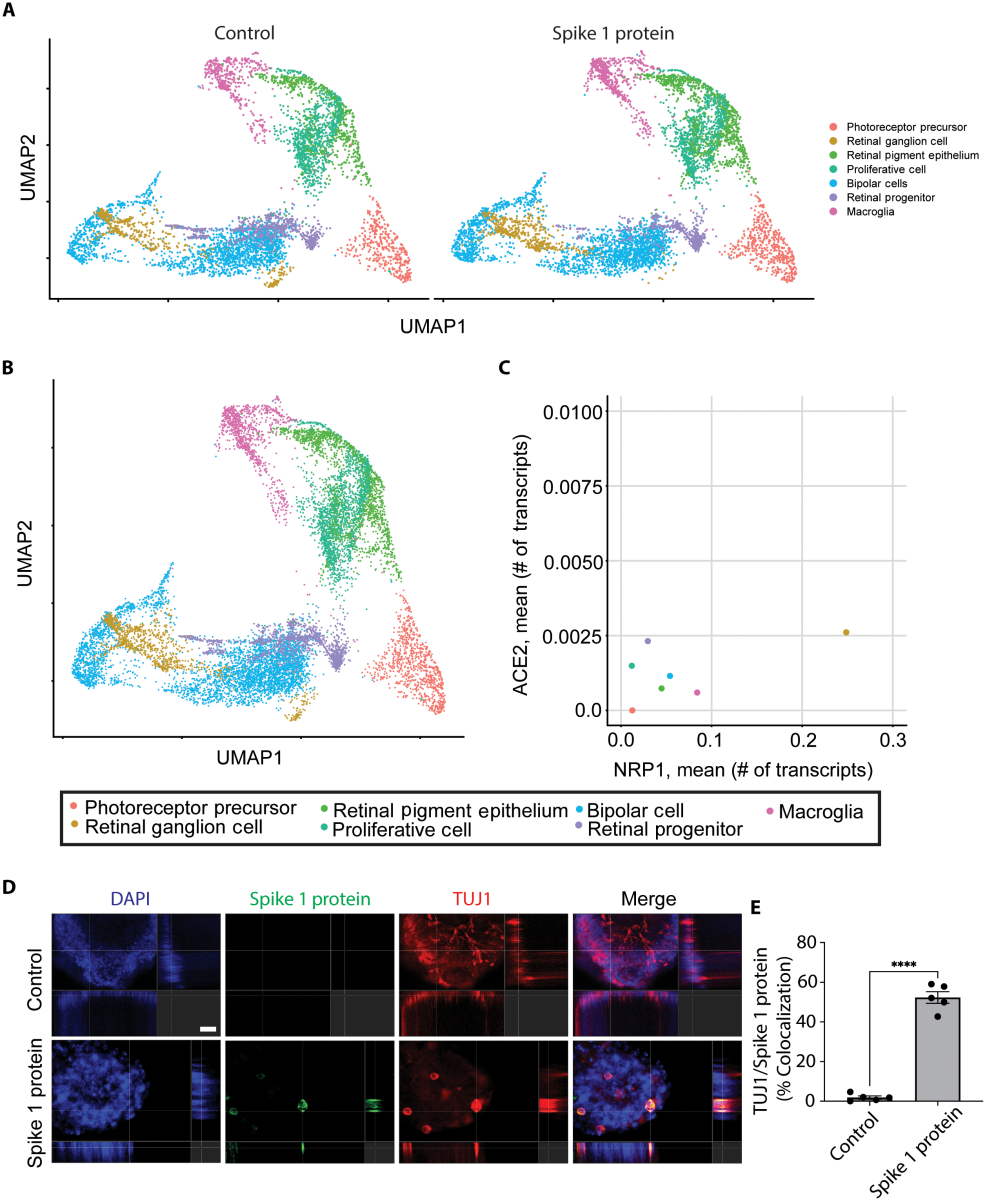
Human retinal organoids are multicellular and bind to SARS-CoV-2 Spike 1 protein. (**A**) scRNA-seq UMAP split by treatment with or without the SARS-CoV-2 Spike 1 protein in control human retinal organoids (*n* = 50 organoids per group). (**B**) Integrated UMAP plot showing clusters of seven distinct cell types in the vehicle control– and Spike 1 protein–treated human retinal organoids. (**C**) Scatterplot depicting the average expression values of NRP1 and ACE2 for each cell type in the vehicle control human retinal organoids using scRNA-seq. (**D**) Human retinal organoids were treated with the vehicle control or SARS-CoV-2 Spike 1 protein. At 24 hours, immunofluorescence imaging of double-positive Spike 1 protein^+^ (green; Alexa Fluor 488–conjugated) and TUJ1^+^ (red) neurons in vehicle control– and Spike 1 protein–treated human retinal organoids; scale bar, 30 μm. (**E**) Quantification of fluorescence colocalization between Spike 1 protein and TUJ1-positive neurons (error bars denote SEM). DAPI, 4′,6-diamidino-2-phenylindole.

Next, we performed single-cell RNA sequencing (scRNA-seq) on human retinal organoids following treatment with SARS-CoV-2 Spike 1 protein and controls. Clustering was performed using the Louvain algorithm and visualized using uniform manifold approximation and projection (UMAP) (Fig. 2, A and B) (*42*). On the basis of characteristic marker expression, the resulting clusters were manually assigned to seven distinct cell types: retinal pigment epithelium, photoreceptor precursors, macroglia, retinal ganglion cells, proliferative cells, retinal progenitors, and bipolar cells (fig. S4A) (*43*, *44*).

To identify the cognate cells for Spike 1 protein binding in the human retinal organoids, we examined the scRNA-seq data for cell type–specific transcript levels of the NRP1 and ACE2 receptors, previously described as involved in SARS-CoV-2 and other viruses to enter neurons (*16*, *18*). Retinal ganglion cells and macroglia showed high expression of NRP1, suggestive of the SARS-CoV-2 Spike 1 protein having affinity for these two cell types (Fig. 2C). We detected expression of ACE2 in neuronal and glial populations, consistent with previous reports (Fig. 2C) (*45*). To show that Spike 1 protein colocalized with human retinal neurons, we performed immunofluorescence with the neuronal marker β-tubulin III (TUJ1) on human retinal organoids treated with the SARS-CoV-2 Spike 1 protein or vehicle control (Fig. 2D) (*46*). At 24 hours, we observed significant colocalization between TUJ1 and Spike 1 protein, further validating the scRNA-seq results (Fig. 2E). Next, using scRNA-seq, we detected transcriptional changes in retinal organoids following SARS-CoV-2 Spike 1 protein treatment (fig. S4B). Last, we performed single-cell pathway analysis (SCPA) and determined the top statistically significant candidate pathways (fig. S4C) (*47*).

### SARS-CoV-2 enhances amyloid-β plaque formation in human retinal models

**Fig. 3.**
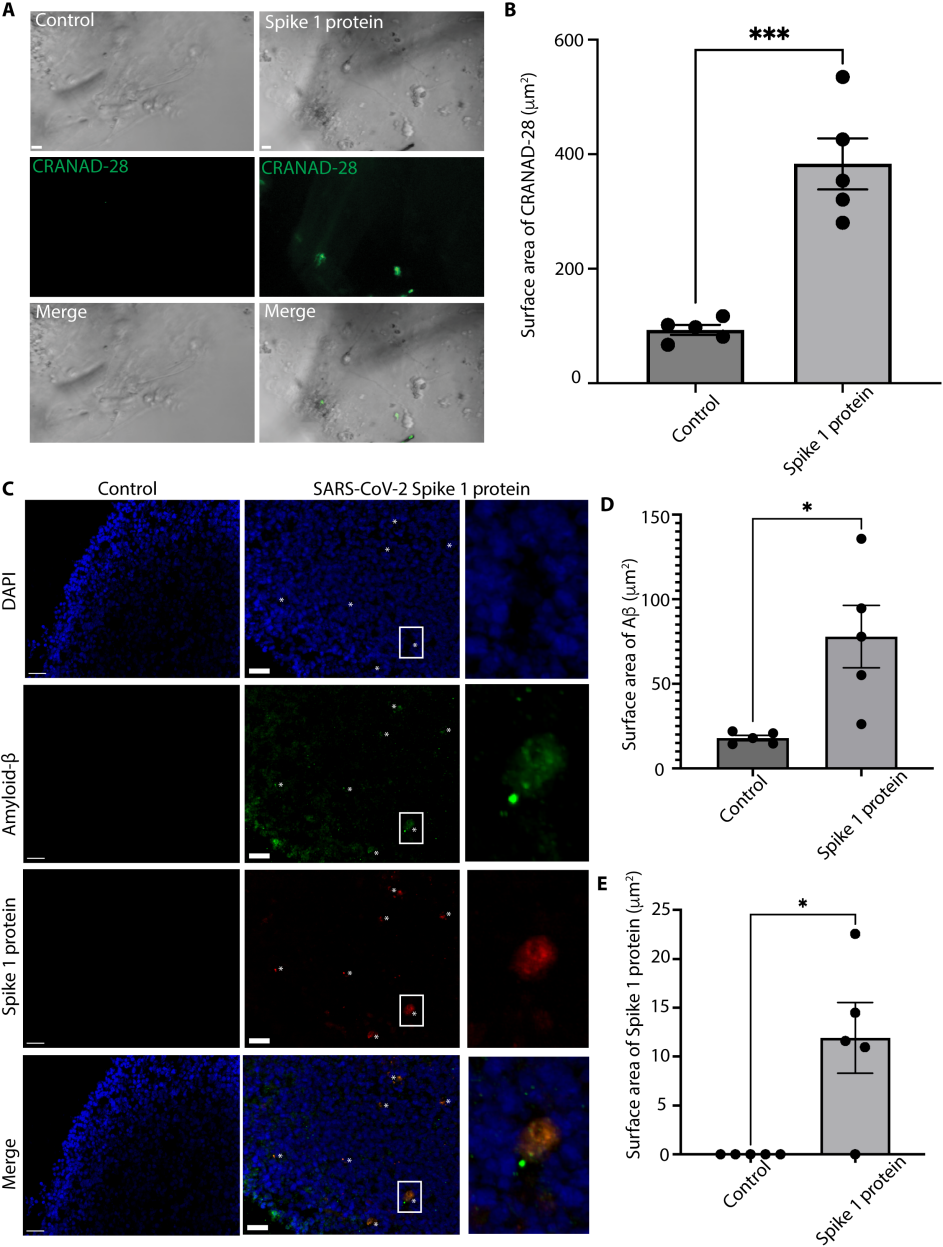
SARS-CoV-2 induces amyloid-β deposition in human retinal organoids. (**A**) Micrographs with bright-field and live-cell fluorescence microscopy with CRANAD-28 in control and Spike 1–treated human retinal organoids (scale bars, 10 μm; *n* = 5). (**B**) Quantification of CRANAD-28 shows increased fluorescence following Spike 1 treatment in human retinal organoids as compared to vehicle controls (*n* = 5; error bars denote SEM). (**C** to **E**) Micrographs and surface area quantification of multiplexed immunofluorescence for amyloid-β (D3E10) and Spike 1 protein (His-tagged) on human retinal organoids with Spike 1 protein or vehicle control (*n* = 5; scale bars, 30 μm; errors bars denote SEM).

To further explore the antimicrobial hypothesis of amyloid-β and its relevance to the COVID-19 pandemic, we treated human retinal organoids with an amyloid-β live-cell imaging curcumin-analog molecule, CRANAD-28, following SARS-CoV-2 Spike 1 protein treatment (Fig. 3A) (*34*, *36*, *37*). There was a statistically significant increase in amyloid-β deposition in the human retinal organoids, as detected by CRANAD-28 after Spike 1 protein treatment compared with controls (Fig. 3B). Immunofluorescence on human retinal organoids treated with the SARS-CoV-2 Spike 1 protein showed increased amyloid-β (D3E10), which colocalized with Spike 1 protein as compared to controls (Fig. 3, C to E) (*48*, *49*). These data indicate that SARS-CoV-2 Spike 1 protein can induce amyloid-β deposition in aged human retinal organoids.

**Fig. 4.**
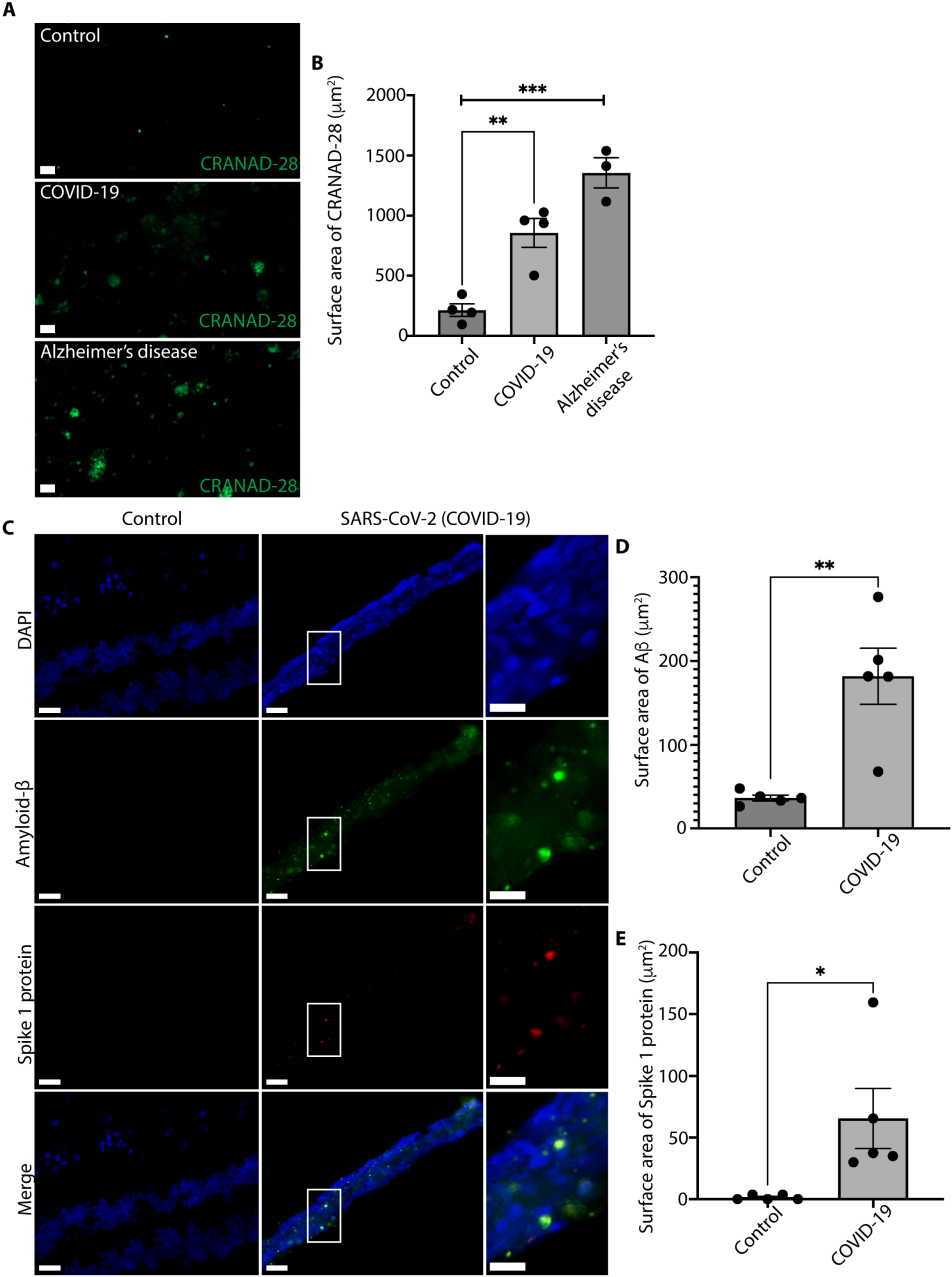
Retinas from short-interval autopsies reveal colocalization of amyloid-β with SARS-CoV-2. (**A**) Micrographs of the human retina stained with CRANAD-28 show elevated amyloid-β in COVID-19 (*n* = 4) and Alzheimer’s disease retinas (*n* = 3) as compared to controls (*n* = 4; scale bars, 20 μm). (**B**) Quantification of CRANAD-28 indicates increased amyloid-β in postmortem human retinal samples with COVID-19 and Alzheimer’s disease as compared to controls (error bars denote SEM; two-tailed Student’s *t* test; **P < 0.01 and ***P < 0.001). (**C** to **E**) Multiplex immunofluorescence for amyloid-β (MOAB-2 antibody) and Spike 1 protein on human retinas from control (*n* = 5) and COVID-19 donors [*n* = 5; scale bars, 50 μm; computational zoom (right) scale bars, 25 μm]. *P < 0.05.

Next, we analyzed postmortem human retinal tissue from patients with COVID-19 [no dementia or mild cognitive impairment (MCI)], those with Alzheimer’s disease, and cognitively normal controls for levels of amyloid-β using CRANAD-28 and an antibody selective for amyloid-β (MOAB-2) (*50*). Compared to control human retinal samples (*n* = 4), there was an increase in CRANAD-28–labeled amyloid-β plaques in the COVID-19 (*n* = 4) postmortem human retinas and those from Alzheimer’s disease retinas as compared to controls (*n* = 3; Fig. 4, A and B). We performed multiplex immunofluorescence with an antibody specific for the SARS-CoV-2 Spike 1 protein and amyloid-β (MOAB-2) on postmortem human retinas with COVID-19 and controls (Fig. 4C) (*26*, *50*, *51*). There were increased levels of amyloid-β in human retinas from donors affected with COVID-19 with no prior history of Alzheimer’s disease or cognitive impairment when compared to control donors (Fig. 4, C to E). A subset of amyloid-β plaques from donors affected with COVID-19 colocalized with SARS-CoV-2 Spike 1 protein (Fig. 4C).

### Single-nucleus RNA-seq identifies transcriptional changes in human COVID-19 retinas

**Fig. 5.**
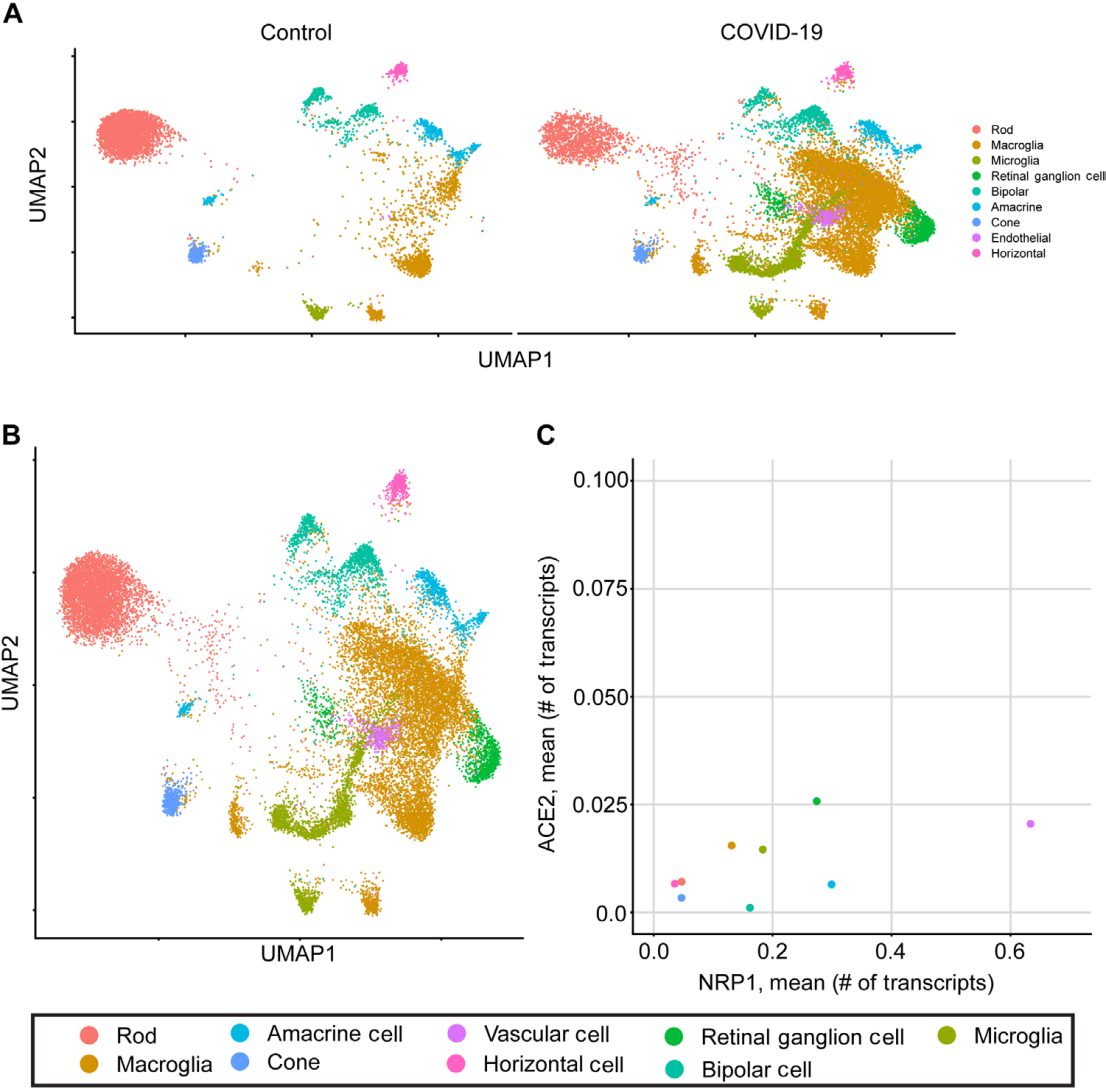
snRNA-seq profiling of human COVID-19 retinas. (**A**) Split by disease UMAPs for the individuals without COVID-19 and with COVID-19, with no medical history of dementia or cognitive impairment. (**B**) Integrated UMAP plot showing clusters of nine distinct cell types in the snRNA-seq data of human retinal samples from control (*n* = 3) and COVID-19 (*n* = 3) donors. (**C**) Scatterplot demonstrates cells expressing ACE2 or NRP1 in snRNA-seq data.

To identify biological pathways affected by SARS-CoV-2 in human retinas, we performed single-nucleus RNA-seq (snRNA-seq) on human retinas from COVID-19 donors (*n* = 3). We integrated these data with age-matched human control retinas (with no reported history of retinopathy, dementia, or cognitive impairment) from previously published work that was sequenced before the COVID-19 pandemic (*n* = 3) (*44*). The snRNA-seq data were clustered and visualized using UMAP (Fig. 5, A and B). On the basis of characteristic marker expression, the clusters were manually assigned to nine distinct cell types: rods, cones, bipolar cells, retinal ganglion cells, horizontal cells, amacrine cells, microglia, vascular cells, and macroglia. Next, we examined the levels of NRP1 and ACE2 expression across retinal cell types and detected both receptors in neuronal, glial, and vascular cell types (Fig. 5C). We detected expression of ACE2 in neurons and glia in postmortem human retinas (Fig. 5C) (*52*). Last, we assessed cell types for the most differentially expressed transcripts and pathways in COVID-19 donors as compared to controls (fig. S5, A and B). The COVID-19 snRNA-seq data indicate expression of the SARS-CoV-2 Spike 1 protein receptors in human retinal cells.

**Fig. 6.**
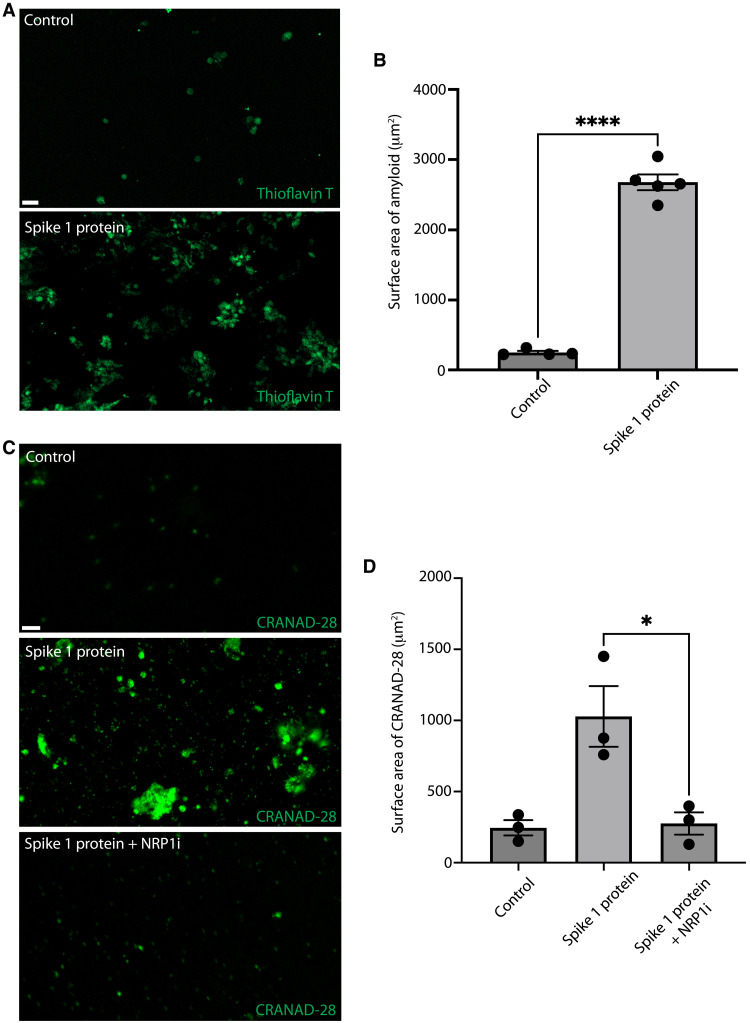
Spike 1 protein–induced amyloidopathy is mediated in part through NRP1. (**A**) Control human retinal samples after 48-hour treatment with phosphate-buffered saline (PBS; control) or Spike 1 protein were stained with thioflavin T to visualize amyloid deposits (*n* = 4). (**B**) Quantification of the surface area with thioflavin T staining between the control and Spike 1 protein–treated groups (*n* = 4). (**C**) Human postmortem retinas were treated with PBS (control) (*n* = 3), Spike 1 protein (*n* = 3), and Spike 1 protein with NRP1 inhibitor (NRP1i; *n* = 3) and amyloid-β plaques visualized with CRANAD-28. (**D**) Quantification of CRANAD-28 surface area in human retinal samples treated with Spike 1 protein and NRP1 inhibitor or vehicle control (*n* = 3). Error bars denote SEM.

Next, to assess the effect of SARS-CoV-2 Spike 1 protein on the human retina, we cultured human retinal explants from rapid autopsies. Human retinas were treated with SARS-CoV-2 Spike 1 protein or control, followed by the application of the amyloid stain thioflavin T to visualize the amyloid burden (Fig. 6A) (*53*). We noted a significant increase in the thioflavin T fluorescence signal, demonstrating increased amyloid deposition in human retinas following SARS-CoV-2 Spike 1 protein exposure (Fig. 6B). These data show that the SARS-CoV-2 Spike 1 protein can induce amyloid deposition in ex vivo human retinas.

### NRP1 inhibitor can inhibit the SARS-CoV-2–induced amyloidopathy

Next, we assessed whether blocking NRP1, a receptor for SARS-CoV-2, can inhibit amyloid deposition following Spike 1 protein treatment. We applied SARS-CoV-2 Spike 1 with and without an NRP1 inhibitor for 24 hours on postmortem human retinal tissue. At 24 hours, the NRP1 inhibitor decreased the accumulation of amyloid-β, as labeled by CRANAD-28, following SARS-CoV-2 Spike 1 treatment as compared to vehicle control (Fig. 6, C and D). These data show that blocking NRP1 can decrease the amyloid-β deposition in human retinas following SARS-CoV-2 Spike 1 treatment.

## DISCUSSION

Here, we demonstrate that SARS-CoV-2 induces CNS amyloid-β deposition in electrophysiologically active ex vivo human retinas from short-interval autopsies and in an accelerated aging human retinal organoid model. Retinas from patients with COVID-19 revealed amyloid-β pathology that colocalized with SARS-CoV-2 Spike 1 protein. Treatment with an NRP1 inhibitor decreased Spike 1 protein–induced amyloidopathy in human retinas. These data suggest that SARS-CoV-2 Spike 1 protein during COVID-19 infection can induce amyloid-β production in the CNS. The accumulation of amyloid-β may be associated with the cognitive symptoms associated with COVID-19 and suggests that NRP1 inhibitors or antiviral medications may serve as therapeutic tools to prevent these consequences.

We speculate that SARS-CoV-2 in the CNS induces a rapid innate immune response. We believe that SARS-CoV-2 works through NRP1 and several other receptors, and once amyloid-β is produced and secreted, it binds to Spike 1 protein with high molecular affinity, which leads to amyloid-β aggregation and encapsulation as a defensive mechanism (*13*, *16*, *19*, *54*). The improper clearance and repeated infections over time may exacerbate the amyloidopathy in Alzheimer’s disease and high-risk groups.

Future studies in humans may help reveal the long-term effects of COVID-19 and the effects of amyloid-β accumulation over time. Investigation of retinal function and amyloid-β using retinal imaging in patients with Long Covid may serve as a novel diagnostic measure of these postsequelae neurological symptoms.

In conclusion, these data suggest that COVID-19 infection, through the SARS-CoV-2 Spike 1 protein, leads to Alzheimer’s disease–related pathology. Inhibition of the cognate receptor for SARS-CoV-2 Spike 1 protein in neurons, NRP1, decreases amyloid-β accumulation in ex vivo human neural tissue. These investigations may provide insight into approaches to prevent the buildup of amyloid-β associated with viral infections. Future studies will be aimed at identifying therapies to promote residual clearance or reduce excessive amyloid-β deposition post–COVID-19 infection. Last, these studies suggest a potential approach to clinically define CNS Long Covid syndromes by measuring retinal function and amyloid-β burden.

## MATERIALS AND METHODS

### Ethics statement

This study and the acquisition and use of postmortem human retinal samples were approved by the Yale Human Research Protection Program’s Institutional Review Board (Yale Protocol Number 2000028616). We complied with all relevant ethical regulations for work with human participants. All human tissue samples were obtained with informed consent before tissue collection from participants, if enrolled antemortem, or legal guardians, if postmortem.

### Human donor samples

Postmortem globes (*n* = 20) and medical records were obtained from the Lions Gift of Sight Eye Bank (Minnesota) and the Yale Tissue Donation Legacy Program from deidentified postmortem donors with informed consent and approval from the Yale Human Subjects Protections committee and with a postmortem interval of <12 hours for live-cell imaging and snRNA-seq and <4 hours for electrophysiological recordings (table S1). Globes were placed in Dulbecco’s modified Eagle’s medium (DMEM; Thermo Fisher Scientific) and transported on ice. Trephine punches (6 mm diameter) were used to isolate peripheral retina samples, which were mechanically separated from the retinal pigment epithelium and choroid layers and transferred to tissue culture wells for retinal explant culture.

### Human retinal organoids treated with and without Spike 1 protein

The “control” was obtained from Yale’s Stem Cell Core (catalog no. Y6), and the New York Stem Cell Foundation’s “Alzheimer’s disease” human iPSCs containing two pathogenic mutations, APP K670_M671delinsNL (Swedish/Swedish) and PSEN1 M146V/M146V (catalog no. BN0002), were differentiated into retinal organoids using a previously established accelerated protocol (*40*). Briefly, human iPSCs were organized into small clumps (~300 μm in diameter) and encapsulated in undiluted Geltrex to form embryoid bodies in mTESR Plus medium. Next, developed embryoid bodies were recovered, plated with a 3:1 mixture of mTESR Plus and neural induction media (DMEM/F12 with l-glutamine, N2 supplement, nonessential amino acids, heparin, Pen-Strep, and GlutaMAX), and annotated as day 0. The following day, the medium was changed daily from 2:1 to 1:1 to become a 100% neural induction medium. Over the duration of 11 days, embryoid bodies are formed into neural spheres that were recovered and suspended in a non–tissue culture–treated plate. On day 11, neural sphere medium is changed to retinal differentiation medium (DMEM/F12 with l-glutamine, B27 supplement, nonessential amino acids, Pen-Strep, and GlutaMAX), with the medium being changed every other day. By day 60, multicellular complex retinal organoids are formed. Day 60 human retinal organoids were treated with phosphate-buffered saline (PBS) for the control condition and Spike 1 protein (10 μg/ml; Sino Biological, catalog no. 40591-V08H14) for the Spike 1 protein condition in BrainPhys medium (STEMCELL Technologies).

### Immunofluorescence imaging

Human retinal samples and/or day 60 human retinal organoids were fixed with 4% paraformaldehyde for 60 min. The paraformaldehyde was removed, and samples were washed three times with PBS. Next, fixed samples were placed in a blocking buffer containing 10% Normal Donkey Serum (Jackson ImmunoResearch Laboratories, 017-000-121) and 0.5% Triton X-100 (Sigma-Aldrich, T8787) for 4 hours at room temperature. The following antibodies against human antigens were used: TUJ1 (1:500; Abcam, catalog no. ab18207), MAP2 (1:500; Abcam, catalog no. ab92434), amyloid-β (6E10, Thermo Fisher Scientific, catalog no. MA5-48043), amyloid-β (H31L21, Invitrogen, catalog no. 3016597), amyloid-β (D3E10, Cell Signaling Technology, catalog no. 12843), amyloid-β (MOAB-2, Abcam, catalog no. Ab126649), Spike 1 (Invitrogen, catalog no. MA5-36247), and His-tagged (Abcam, catalog no. Ab18184) for 24 hours in PBS at 4°C. After washing three times with PBS, secondary staining was performed using donkey antirabbit (488AF; 1:1000; Invitrogen, catalog no. A21206) for 8 hours at room temperature. Next, retinal samples were washed three times with PBS and placed into optical well plates (Azenta Life Sciences) for microscopy. Imaging was performed using a confocal microscope (Zeiss LSM800, Jena, Germany).

### Electrophysiology and two-photon microscopy

Patch-clamp recordings were made from short-interval autopsy postmortem human retinal explants in flat-mount and whole-spherical organoids held down to the bottom of a recording chamber by a nylon mesh glued on a platinum ring (*n* = 2). The tissues were superfused in the recording chamber with artificial cerebrospinal fluid (120 mM NaCl, 3.1 mM KCl, 1.1 mM CaCl_2_, 1.2 mM MgCl_2_, 1.25 mM MgSO_4_, 26 mM NaHCO_3_, 0.5 mM l-glutamine, 0.1 mM ascorbic acid, 0.1 mM Na-pyruvate, and 20 mM glucose) saturated with 95% O_2_ to 5% CO_2_ at 32° to 34°C. The pipette solution used for current clamp contained 105 mM k-gluconate, 5 mM KCl, 2 mM EGTA, 10 mM Na_2_-phosphocreatine, 2 mM Mg–adenosine triphosphate (ATP), 0.5 mM Na_2_–guanosine triphosphate (GTP), 2 mM ascorbic acid, and 10 mM Hepes (pH 7.2 with KOH). Pipette solution for voltage clamp contained 110 mM CsMeSO_4_, 5 mM NaCl, 0.5 mM CaCl_2_, 2 mM MgCl_2_, 5 mM EGTA, 2 mM ATP-2Na, 0.5 mM GTP-2Na, 2 mM ascorbic acid, and 10 mM Hepes (pH 7.2 with 20 to 30 mM CsOH). Alexa Fluor 594 was included in pipette solutions for cell labeling. TTX (1 μM) was applied by bath perfusion (2 to 4 ml/min). Ganglion cells in flat-mounted postmortem human retinal explants (ganglion cell side facing upward) were recorded under an upright microscope (BX51, Olympus, New York) configured with infrared differential interference contrast optics and a two-photon imaging system (Ultima, Bruker Nano Inc., Madison, WI, USA). Cells on the outermost surface of the organoid were targeted for recording under differential interference contrast optics of an upright microscope (BX50, Olympus). Electrophysiological data were recorded with a Multiclamp 700A (or 700B) patch-clamp amplifier (Molecular Devices, Sunnyvale, CA), stored on Power Lab (AD Instruments, Colorado Springs, CO), and analyzed with pClamp10 (Molecular Devices) and Origin 9 Software (Origin Lab Corp., Northampton, MA). Liquid junction potentials were calculated and corrected. Current-voltage relations were plotted after membrane leak subtraction.

After patch-clamp recording, the morphology of recorded cells was reconstructed from *z* stacks of two-photon images acquired under a two-photon imaging system using an 870-nm laser (Ti:sapphire tunable pulsed laser, MaiTai, Newport, CA) with a 60×, 1.0 numerical aperture objective (LUMPlanFL/IR, Olympus USA). Fluorescence signals were band-pass filtered at 607 ± 23 nm (Chroma HQ607/45, green channel) and 520 ± 18 nm (Semrock FF0-520/35-25, red channel) and collected by two photomultiplier tubes, respectively. Acquired image stacks were analyzed with ImageJ (NIH, Bethesda, MD, USA).

### Live-cell imaging of human retinal organoids

We treated human retinal organoids with the live-cell imaging small molecule, CRANAD-28 (0.1%; from C. Ran), to visualize amyloid-β plaques (M5000, Thermo Fisher Scientific). The green fluorescent protein (GFP) channel was used to visualize the CRANAD-28 at 24 hours posttreatment. Micrographs (*n* = 5 organoids per group) were saved as .TIFF files and imported into Oxford’s Imaris Software. Imaris surface rendering was performed on the GFP channel of the retinal organoid micrographs, and the resulting data were exported into a .csv file with the sum area of CRANAD-28 fluorescence being quantified and displayed.

### Live-cell imaging of human retina with and without NRP1 inhibitor

The human retinal explants were treated with Spike 1 protein (10 μg/ml) or vehicle control. Treatments were added to DMEM/F12 (1:1) with l-glutamine and 2% penicillin-streptomycin daily, with complete medium changes. The human retina was then subjected to live-cell imaging with thioflavin T (1:250) or CRANAD-28 (0.1%; from C. Ran) for 10 min and then to micrographs taken with confocal microscopy (Zeiss LSM800). For NRP1 inhibitor (Apex Bio, catalog no. B4657) treatment, 8.5 μM was applied at the same time as Spike 1 protein and control treatments. For human retinal live-cell imaging, we treated with 0.1% CRANAD and imaged on the GFP channel.

### Image processing and analysis

Oxford Imaris Software was used for all image analysis with the following programs: spot detection, surface rendering, and batch processing of multiple samples.

### scRNA-seq of human retinal organoids

Day 60 human retinal organoids were obtained from low-attachment cell culture plates and subjected to dispase II dissociation solution according to the manufacturer’s protocol. Briefly, 50 organoids were dissociated into single-cell suspensions, and Chromium Next GEM Single Cell 3′ Reagent Kits v3.1 were used to assemble RNA libraries of single cells. The organoids were batched per treatment (S1-treated and control groups) and subjected to partial lane sequencing on the Illumina NovaSeq 6000. The raw data obtained from the sequencing were converted into FastQ files, which were then aligned to the human reference genome (GRCh38) using the 10X Cell Ranger 7.1.0 pipeline.

### Human amyloid-β42 ELISA

Isolated postmortem human eyes were obtained from Alzheimer’s disease (*n* = 3) and control (*n* = 3) donors and lysed with Tissue Protein Extraction Reagent plus phosphatase inhibitors (Thermo Fisher Scientific) or human retinal organoids (*n* = 20) that were harvested from the Y6 or double pathogenic line received from the New York Stem Cell Foundation. Human amyloid-β42 ELISAs were performed according to the manufacturer’s protocol (Abcam, catalog no. Ab289832; FUJIFILM Wako, catalog no. 296-6440).

### Hematoxylin and eosin human retinal staining

Human eye globes were embedded into cryo-preserving medium, snap frozen with liquid nitrogen, and stored at −80°C. The following day, human eye globes were sectioned (10 μM) and stained with the hematoxylin and eosin protocol according to the manufacturer’s instruction (Abcam, catalog no. ab245880).

### Processing, cleaning, and integrating scRNA-seq data

The raw 10X Genomics–filtered matrix files were processed using Seurat (*42*). Quality control measures to trim poor-quality data were applied separately to the vehicle control– and Spike 1–treated datasets before integrating them and reducing data dimensionality.

For the initial filtering step, only cells with a minimum of 200 detected genes and genes detected in at least three cells were included. In the Spike 1 protein–treated group, cells were further filtered on the basis of cutoffs for unique genes (200 to 7000), unique molecular identifiers (UMIs) (100 to 20,000), mitochondrial DNA percentage (0 to 30%), and ribosomal percentage (5 to 45%). In the control group, the cutoffs were unique genes (200 to 6100), UMIs (100 to 21,000), mitochondrial DNA percentage (0 to 35%), and ribosomal percentage (0 to 50%).

Following the initial filtering, the data underwent dimensionality reduction. First, log normalization was applied to minimize noise and bias introduced during data production. Next, a linear transformation was applied to center the mean gene expression to 0 and set the variance to 1. Principal components analysis (PCA) was then performed on the data, and it included 2000 highly variable genes. Subsequently, the data were integrated using Harmony, resulting in a final number of 50 dimensions.

The unsupervised, hierarchical Louvain algorithm was used for clustering the cells using a modularity resolution limit of 0.3. To visualize the data, the Seurat RunUMAP function was used to generate a UMAP plot using the first 50 principal components. Cells belonging to a previously labeled “Unknown” cluster, characterized by notably low RNA features compared to other clusters, were excluded from the final Seurat object and UMAP. On the basis of characteristic marker expression, the remaining cells comprised seven clusters, which were assigned to seven distinct retinal cell types: retinal pigment epithelium, photoreceptor precursor, macroglia, retinal ganglion cells, proliferative cell, retinal progenitor, and bipolar cell.

### Nuclei isolation

To perform snRNA-seq on retinal samples from donors with COVID-19 (*n* = 3), nuclei were isolated from the tissue using the Nuclei Isolation Kit Nuclei EZ Prep (Sigma-Aldrich, NUC-101). The tissue sample and 1 ml of the provided lysis buffer were put into a 2-ml Dounce tissue grinder, after which they were manually homogenized 25 times with a pestle having a wide clearance gap. Next, an additional 1 ml of lysis buffer was added, and the mixture was further homogenized 25 times with a pestle having a narrow clearance gap. The resulting lysate was transferred to a 15-ml conical tube along with 2 ml of lysis buffer, after which they were mixed, incubated on ice for 5 min, and placed in a swinging-bucket centrifuge at 500*g* and 4°C for 5 min. The supernatant was then removed, and the remaining pellet was resuspended in 4 ml of lysis buffer and mixed, incubated, and centrifuged under the same conditions as previously used. The supernatant was again aspirated, and the remaining pellet was washed in 4 ml of nuclei suspension buffer (1× PBS containing 0.01% bovine serum albumin and 0.1% ribonuclease inhibitor) before undergoing another round of incubation, centrifugation, and aspiration. The resulting pellet was resuspended in 1 ml of nuclei suspension buffer and passed through a 40-μm nylon filter. Nuclei were manually counted on a hemocytometer (C-Chip, SKC Inc., DHCN015) with trypan blue, and the final nuclei suspension was diluted to target a recovery of 10,000 nuclei in the library preparation.

### snRNA-seq of human retina

RNA libraries of single nuclei were assembled in compliance with the 10X Chromium Next GEM Single Cell 3′ platform’s manufacturer procedures. From retinal explant tissue belonging to the COVID-19 donors, Chromium 3′ v3.1 kits were used to create libraries that were then sequenced using paired-end sequencing on the Illumina NovaSeq 6000. The raw data obtained from the sequencing were converted into FastQ files, which were then aligned to the human reference genome (GRCh38) using the 10X Cell Ranger 7.1.0 pipeline.

### Processing, cleaning, and integrating snRNA-seq data

To perform a comparative analysis across conditions, three previously sequenced control human retina snRNA-seq samples from donors who never had COVID-19 were integrated with the COVID-19 human retina snRNA-seq sample (*27*). For each sample, the filtered feature-barcode matrices obtained from the Cell Ranger 7.1.0 pipeline output were used to create the integrated Seurat object for downstream analysis. For quality control, nuclei were filtered to only include those with unique feature counts between 350 and 3000 and less than 5% mitochondrial counts. The Seurat NormalizeData and FindVariableFeatures functions were then used to normalize the data and identify 2000 features that exhibit high cell-to-cell variation, respectively. Next, the Seurat ScaleData and RunPCA functions were used to scale the data and perform PCA for the first 30 principal components. Last, the Seurat FindNeighbors and FindClusters functions were used to cluster the nuclei, and the Seurat RunUMAP function was used to visualize the data and generate a UMAP plot using the first 30 principal components. On the basis of characteristic marker expression, the remaining cells comprised 35 clusters, which were assigned to nine distinct retinal cell types: rods, cones, bipolar cells, retinal ganglion cells, horizontal cells, amacrine cells, microglia, vascular cells, and macroglia.

### Differential gene expression analysis

Using the Seurat FindMarkers function, differentially expressed genes were found between the Spike 1 protein–treated and control retinal ganglion cells in the scRNA-seq data by setting “ident.1” to the identity of the Spike 1 protein–treated retinal ganglion cells and “ident.2” to the identity of the vehicle control retinal ganglion cells. This same method was applied for the snRNA-seq data to find differentially expressed genes between the COVID-19 and control retinal ganglion cells in the snRNA-seq data by setting ident.1 to the identity of the COVID-19 retinal ganglion cells and ident.2 to the identity of the control retinal ganglion cells. A cutoff of adjusted *P* < 0.05 was used to select only the statistically significant differentially expressed genes.

### Biological pathway analysis

Pathway analysis was conducted using the SCPA toolkit within R (*47*). All Gene Set Enrichment Analysis (GSEA) Hallmark, Kyoto Encyclopedia of Genes and Genomes (KEGG), and Reactome gene sets were loaded using the msigdbr package. Two configurations were run: one for human retinal organoids and one for human retinal explants. For the retinal organoid system, a downsampling parameter of 1000 was used (vehicle control– versus Spike 1 protein–treated), and SCPA was run. The qval and rank of pathways were plotted, and selected pathways were highlighted. For the explant system, SCPA was performed with identical configuration settings, and qval and rank were similarly plotted.

### Statistical analysis

Unpaired, two-tailed *t* test was used to determine the association between the SARS-CoV-2 Spike 1 protein–treated and control groups. Statistical significance was determined by *P* < 0.05. For multiple comparisons, one-way analysis of variance was performed, and significance was denoted as *P* < 0.05. All statistical illustrations were generated by GraphPad Prism software. All error bars are the result of the SEM or SD, as indicated.
