# Supplementary material for: SARS-CoV-2 induces Alzheimer’s disease–related amyloid-β pathology in ex vivo human retinal explants and retinal organoids
Source: Sci Adv. 2025 Jul 4;11(27):eads5006. doi: 10.1126/sciadv.ads5006 (PMC12227045; doi:10.1126/sciadv.ads5006)
Supplement: Supplementary file 1 — Figs. S1 to S6 Table S1 [file sciadv.ads5006_sm.pdf]

Science Advances
AAAS

Supplementary Materials for

SARS-CoV-2 induces Alzheimer's disease–related amyloid-β pathology in ex vivo human retinal explants and retinal organoids

Sean J. Miller *et al.*

Corresponding author: Brian P. Hafler, brian.hafler@yale.edu

Sci. Adv. **11**, eads5006 (2025)
DOI: 10.1126/sciadv.ads5006

This PDF file includes:

Figs. S1 to S6
Table S1

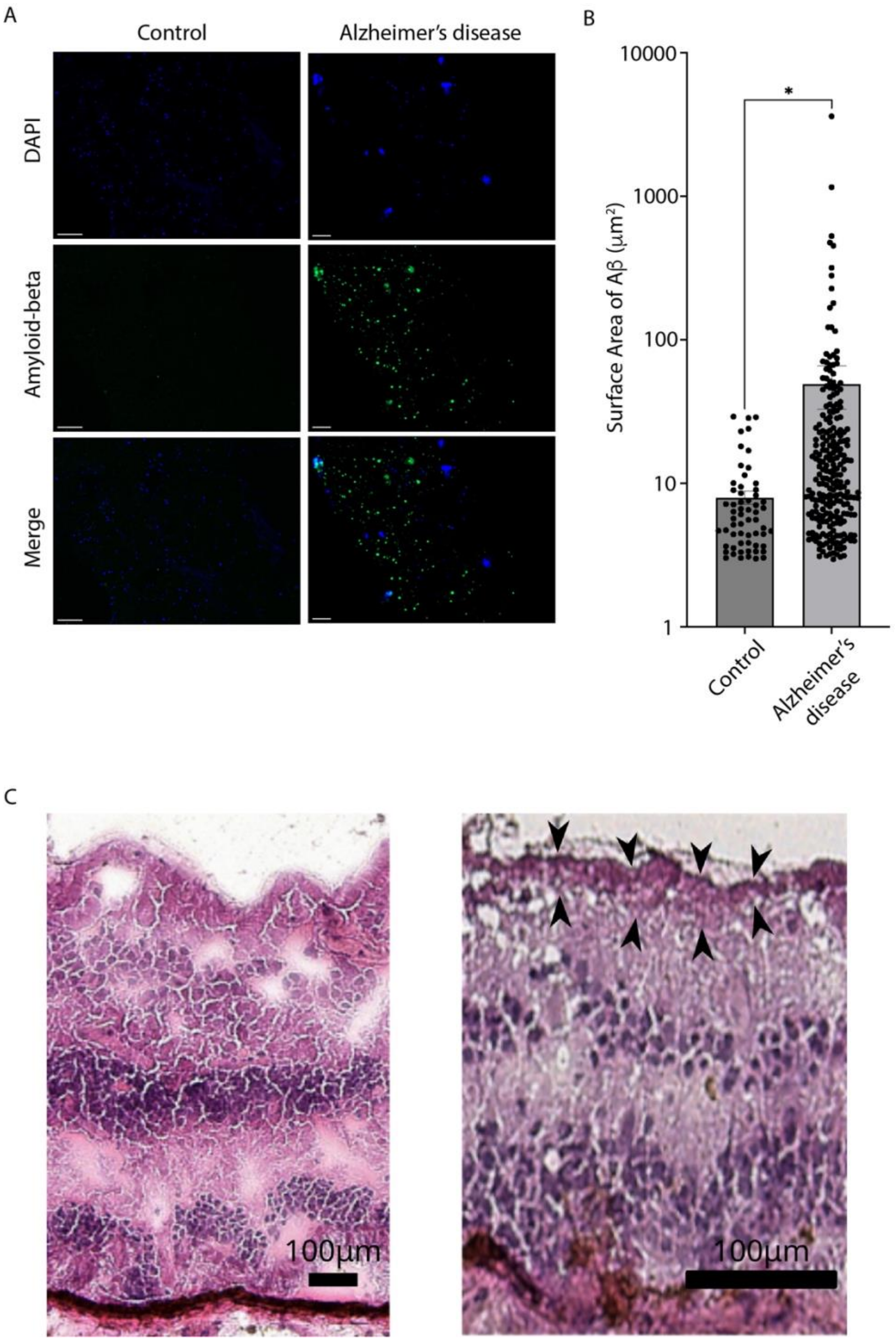

Fig. S1. Human retinas with Alzheimer's disease contain elevated amyloid-beta and astrogliosis of the nerve fiber layer. (A) Micrographs of amyloid-beta immunofluorescence demonstrate elevated levels of amyloid-beta in retinas with Alzheimer's disease compared to age-matched controls (n = 10; scale bars = 30 μM). (B) Quantification of the amyloid-beta fluorescence in log10 using the antibody 6E10 shows statistically significantly higher levels of amyloid-beta in Alzheimer's disease retinas compared to controls

(error bars = SEM). (C) H&E staining of human retinas of control (left) and Alzheimer's disease (right), arrows indicate astrogliosis in the nerve fiber layer.

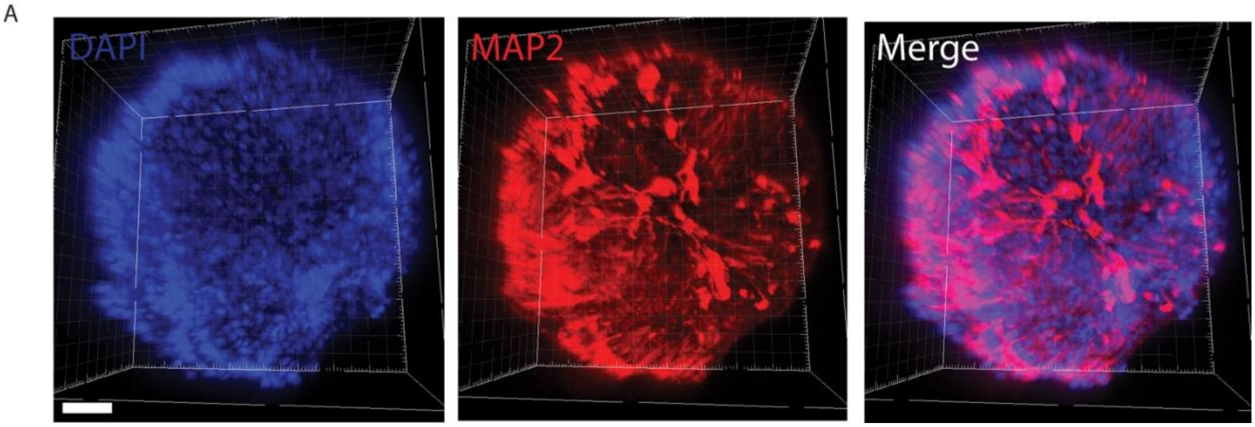

Fig. S2. Human retinal organoids display accelerated neuronal differentiation. (A) Control human retinal organoids at day 60 were stained with MAP2 (red) to label retinal neurons and DAPI (blue) to label cell nuclei (scale bars = 50 μM).

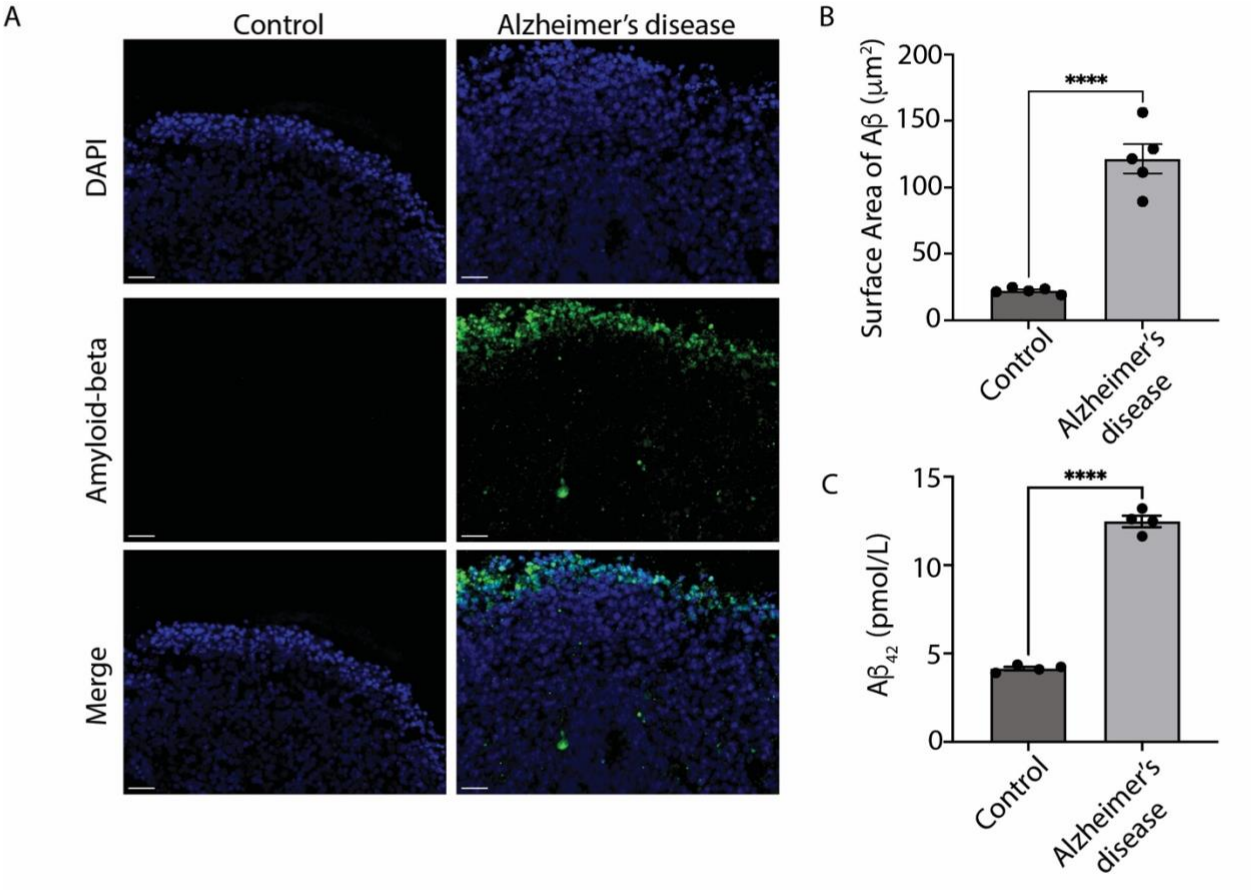

Fig. S3. Human retinal organoids derived from Alzheimer's disease human iPSC contain amyloid beta. (A) Micrographs from Day 60 human control retinal organoids and those derived from iPSCs with

Alzheimer's disease show increased amyloid-beta in Alzheimer's disease retinal organoids using the amyloid-beta antibody, H31L21. (B) Quantification of the surface area of H31L21 (n = 10, error bars = SEM). (C) Human amyloid-beta (42) ELISA (FUJIFILM) was used to determine levels in control retinal organoids (n = 4) and retinal organoids derived from Alzheimer's disease human iPSCs (n = 4; error bars = SEM).

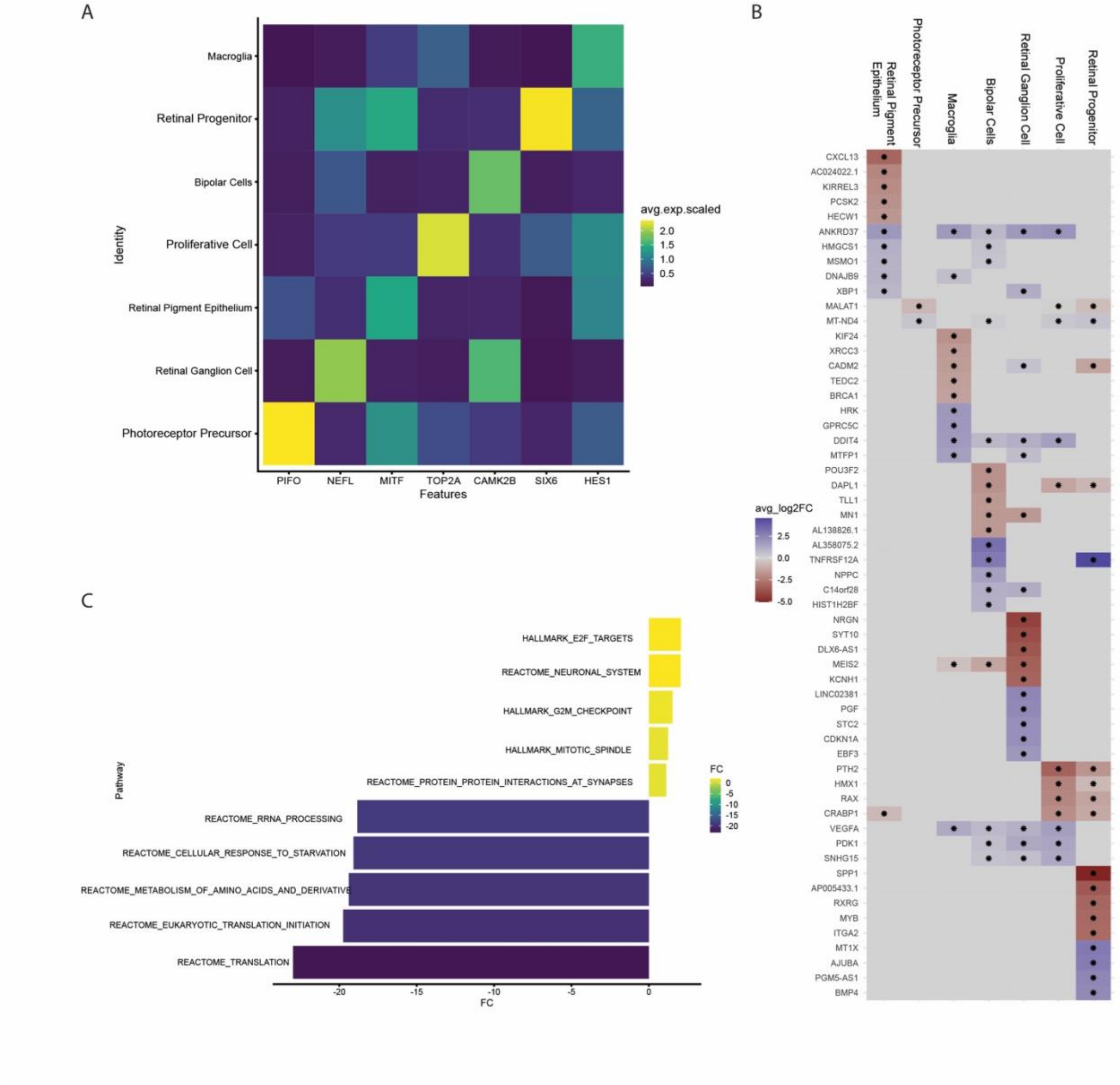

Fig. S4. Human retinal organoids are multicellular and differentially regulate transcription after SARS-CoV-2 Spike 1 protein treatment. (A) Heatmap illustrates 7 cellular clusters in Day 60 human retinal organoids. (B) Gene expression of the top differentially expressed transcripts per cellular cluster

(*Bonferroni-adjusted $p < 0.05$). (C) Top statistically different pathways as determined by SCPA (FC = fold-change).

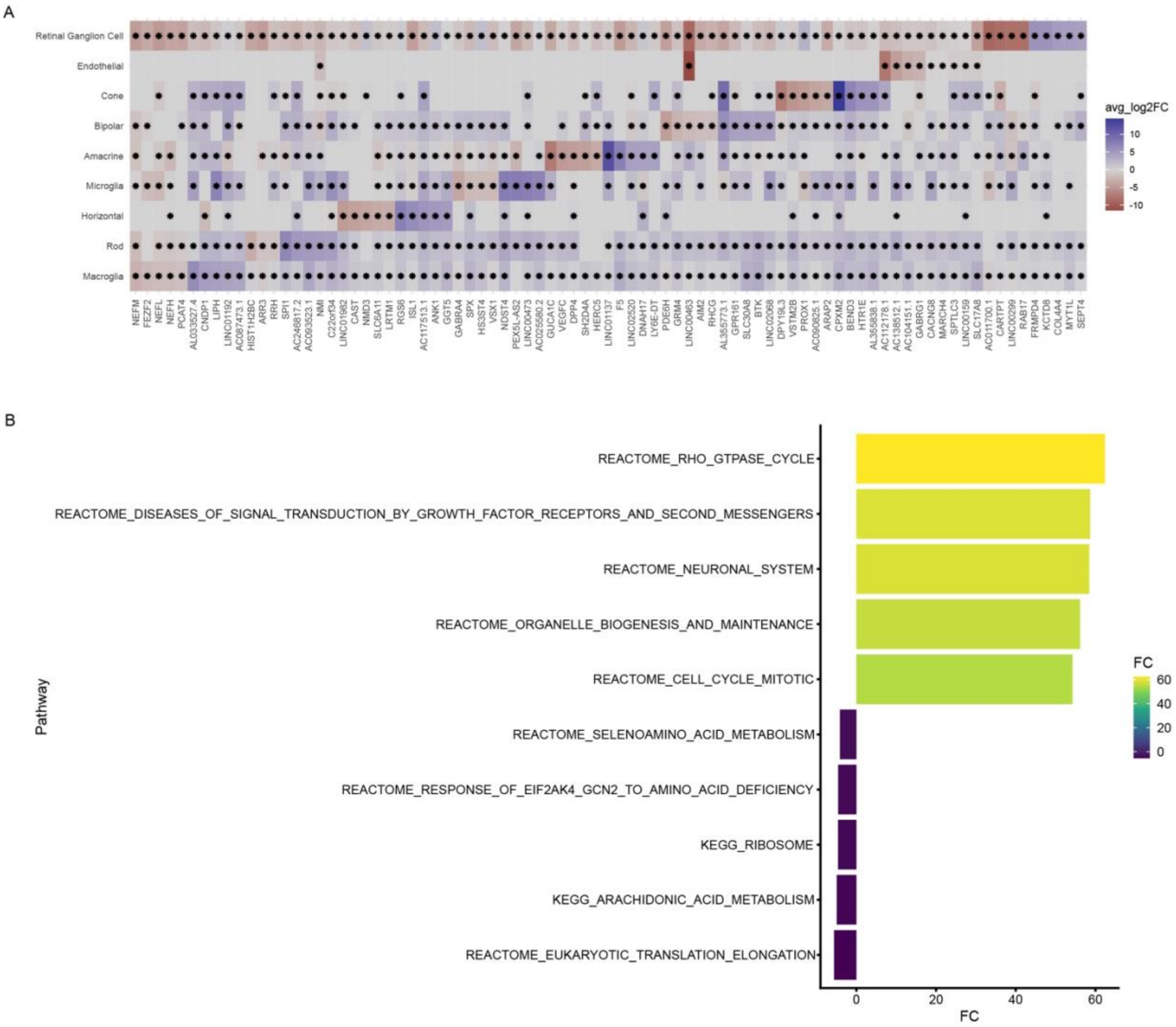

Fig. S5. snRNA-seq of enriched markers and altered pathways between COVID-19 and control retinas. (A) Gene expression of the top differentially expressed transcripts per cellular cluster

(*Bonferroni-adjusted p < 0.05). (B) Top statistically different pathways as determined by SCPA (FC = fold-change).

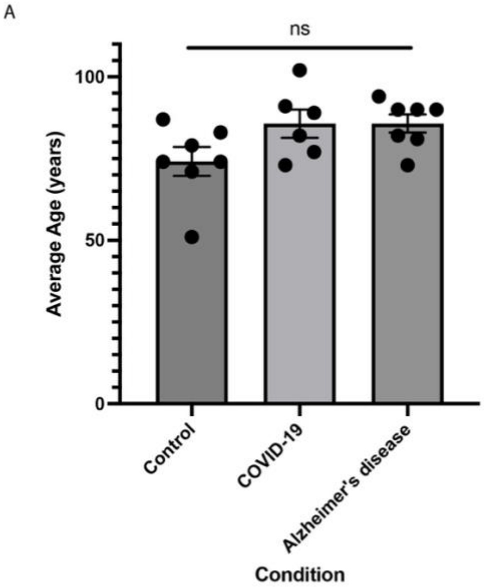

Fig. S6. Age distribution of human retinas obtained from short-interval autopsies. (A) Age per condition shows no significant difference (one-way ANOVA; p > 0.05, errors bars = SEM).

Table. S1. Patient demographics.

**Characteristics**	**Control**	**COVID-19**	**Alzheimer's disease**
**Total patients**	7	6	7
**Sex**			
Male	2	2	3
Female	5	4	4
**Age (years)**			
Median	74	85	90
Range	51-87	73-102	73-94
